# Supplementary figures and images for: Development of a new cell isolation device FlowMagicTM
Source: PLoS One. 2025 Oct 22;20(10):e0334936. doi: 10.1371/journal.pone.0334936 (PMC12543197; doi:10.1371/journal.pone.0334936)

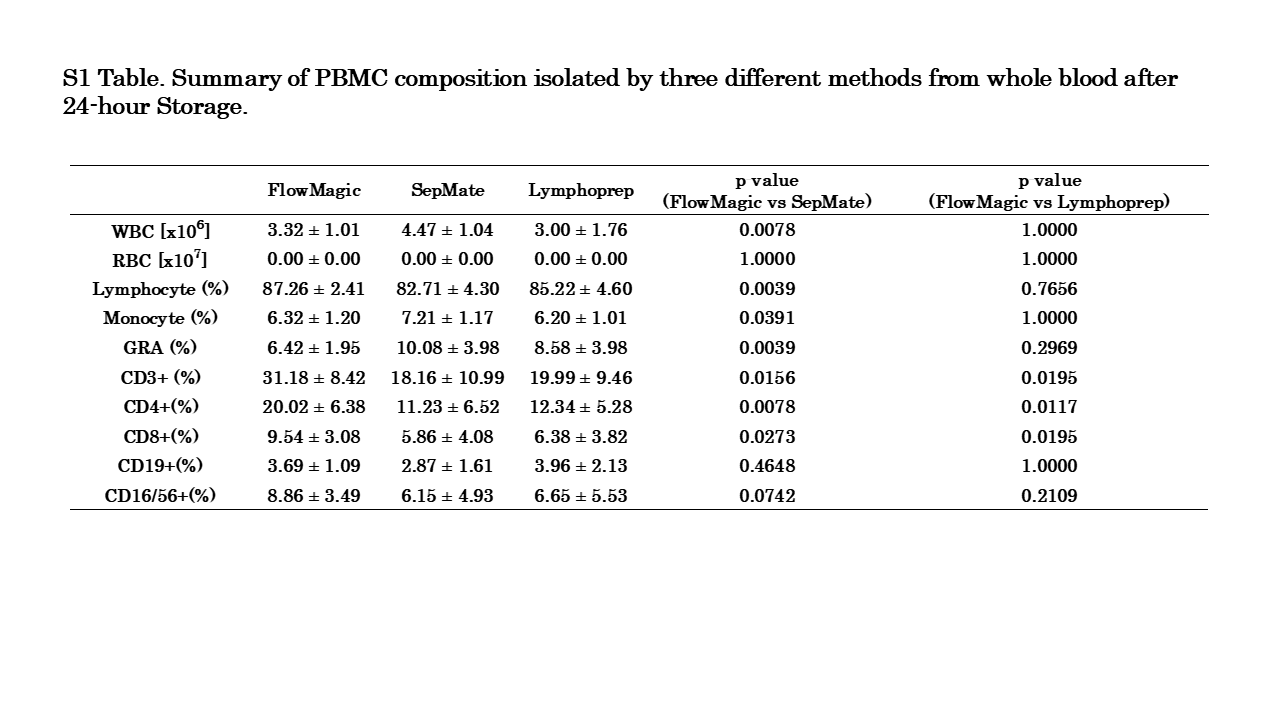

Supplement: S1 Table — Data from 10 volunteers. Values are shown as means ± standard deviation. The Wilcoxon signed-rank test was used with Bonferroni correction for multiple comparisons as a non-parametric alternative. A significance level of 0.05 was used for all statistical tests. PBMC: peripheral blood mononuclear cell; WBC: white blood cell; RBC: red blood cell; GRA: granulocytes. (TIF) [file pone.0334936.s001.tif]

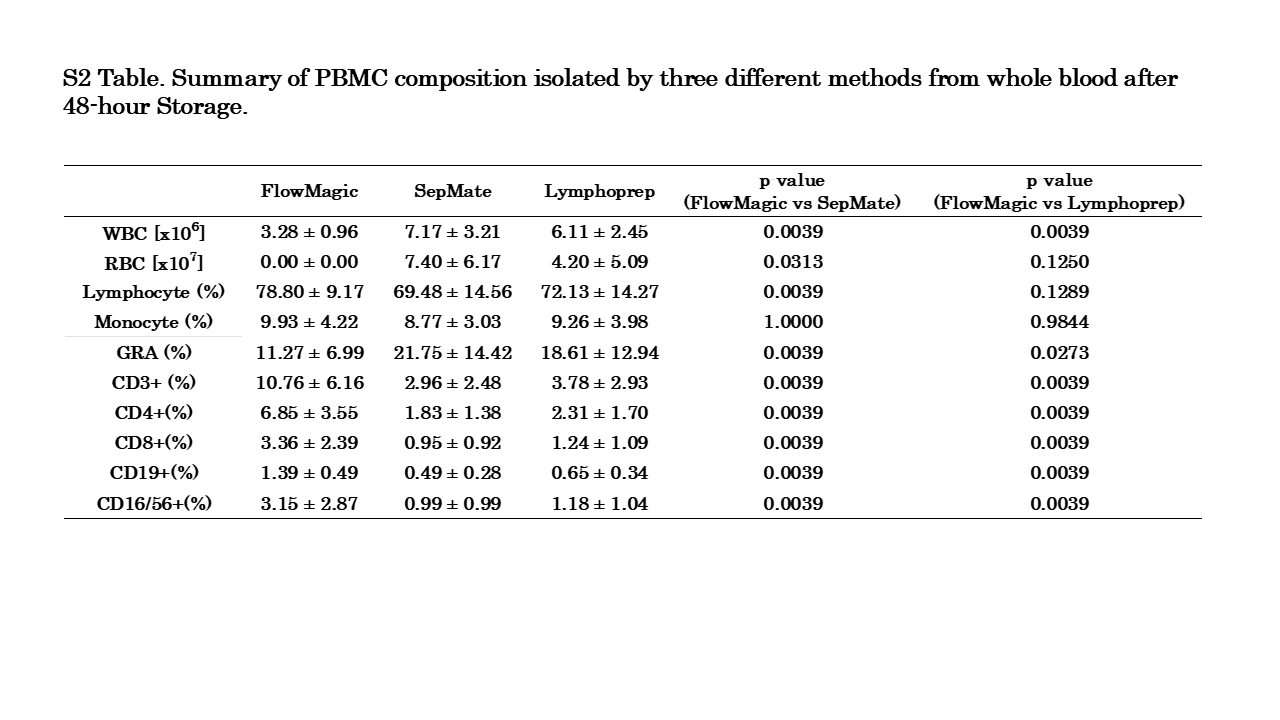

Supplement: S2 Table — Data from 10 volunteers. Values are shown as means ± standard deviation. The Wilcoxon signed-rank test was used with Bonferroni correction for multiple comparisons as a non-parametric alternative. A significance level of 0.05 was used for all statistical tests. PBMC: peripheral blood mononuclear cell; WBC: white blood cell; RBC: red blood cell; GRA: granulocytes. (TIF) [file pone.0334936.s002.tif]

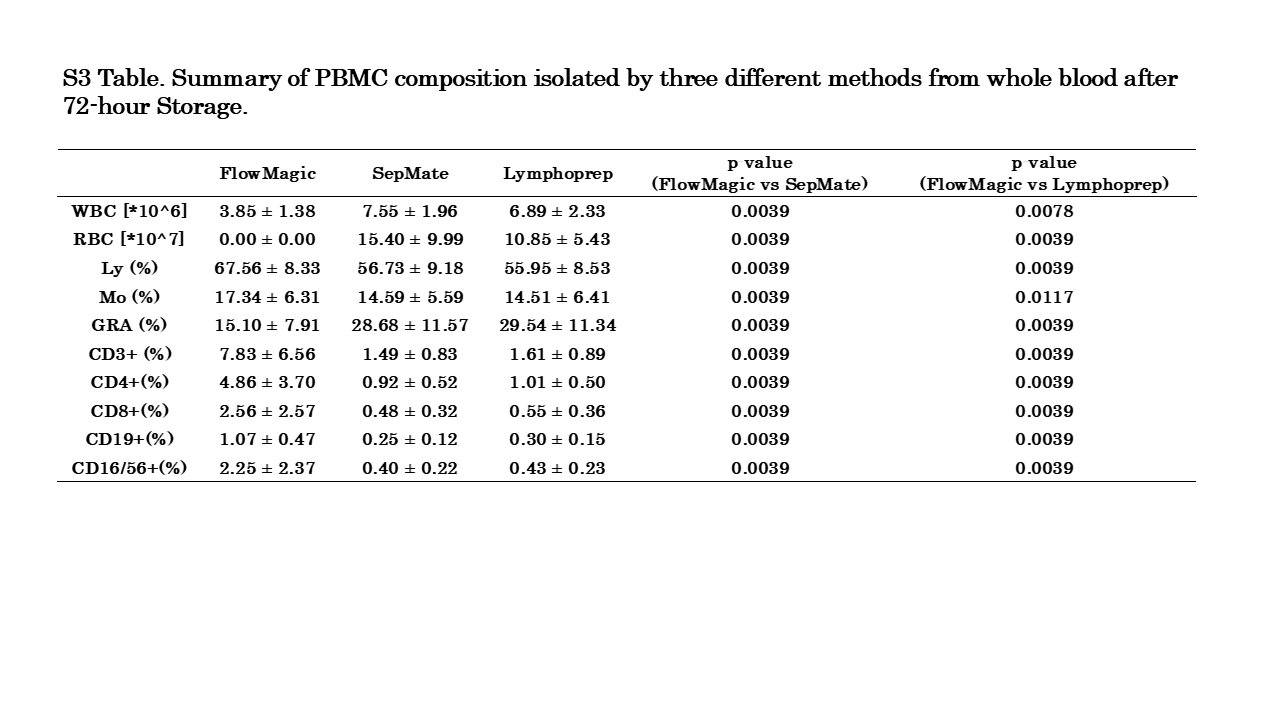

Supplement: S3 Table — Data from 10 volunteers. Values are shown as means ± standard deviation. The Wilcoxon signed-rank test was used with Bonferroni correction for multiple comparisons as a non-parametric alternative. A significance level of 0.05 was used for all statistical tests. PBMC: peripheral blood mononuclear cell; WBC: white blood cell; RBC: red blood cell; GRA: granulocytes. (TIF) [file pone.0334936.s003.tif]
