# Supplementary material for: Development of a new cell isolation device FlowMagicTM
Source: PLoS One. 2025 Oct 22;20(10):e0334936. doi: 10.1371/journal.pone.0334936 (PMC12543197; doi:10.1371/journal.pone.0334936)
Supplement: Attachment — Submitted filename: renamed_cd3c8.docx [file pone.0334936.s004.docx]

This document is a translated version and not the original.

Ethics Review Result Notification

Review Date: February 4, 2025

Expedited Review

To the Research Director

H.U. Group Institutional Review Board

Management Number: 24-015-02 Research and Development

Application Subject: Development of Structure and Device to Enhance Separation Effect

H.U. Group Institutional Review Board has made the following review decision regarding the above-mentioned subject that was submitted on December 24, 2024, and hereby provides notification.

Review Result: Approved

Recommendation: None in particular.
