# Supplementary material for: Development of a new cell isolation device FlowMagicTM
Source: PLoS One. 2025 Oct 22;20(10):e0334936. doi: 10.1371/journal.pone.0334936 (PMC12543197; doi:10.1371/journal.pone.0334936)
Supplement: Attachment — Submitted filename: 250908 Rebuttal letter for Reviewer.docx [file pone.0334936.s005.docx]

**Rebuttal letter**

**Response to reviewer 1**

Reviewer #1: This manuscript addresses an important and timely topic—developing a novel centrifugation-based technique to isolate viable PBMCs from whole blood with minimal WBC and RBC contamination. The authors provide a clear description of current commercial techniques, particularly density gradient centrifugation, and highlight the limitations associated with these existing methods. The manuscript compares the performance of the proposed method against two widely used commercial technologies and includes preliminary data suggesting improved performance. However, several major revisions are necessary before the manuscript can be considered for publication.

1. The authors state that their method performs better than the current technologies, but the abstract and conclusion sections do not include any quantitative data to support this claim. Specific numerical results should be presented to demonstrate the comparative performance clearly.
Reply: We thank the reviewer for their comment. We have included specific numerical data, presenting median values with corresponding interquartile ranges, to provide clear evidence of comparative performance (Fig. 4-6, Table S1-S3). The results demonstrate that FlowMagic™ achieved significantly better outcomes than both SepMate and Lymphoprep methods, with statistical significance established at *p* < 0.01. (Line: 23-38)

2. The conclusions are somewhat overstated given the scope and depth of the current data. The authors should discuss in more detail the limitations of their study and future research that should be conducted.
Reply: We thank the reviewer for this valuable suggestion. We have addressed the above issue in the abstract section of our manuscript. We have revised it as follows: These findings indicate that the PBMC isolation method using FlowMagic™ is advantageous in the isolation process for research and laboratory tests to prevent contamination by RBCs and GRAs. (Lines: 39-41)

3. While the manuscript provides some quantitative data, there is no statistical analysis presented to validate the claim that their technology is superior. Mean values and standard deviations should be reported for all comparative data, and appropriate statistical tests (e.g., ANOVA, t-tests) should be used to evaluate the significance of differences between groups.
Reply: We increased the sample size to 10. For the data from a total of ten volunteers presented in Figures 4, 5, and 6, the Wilcoxon signed-rank test was used with Bonferroni correction for multiple comparisons as a non-parametric alternative. All data were visualized using box plots. A significance level of 0.05 was used for all statistical tests. In response to your recommendation, we have included detailed summary tables in the Supporting Information (Tables S1-S3) that present mean values ± standard deviation. (Figs 4, 5, and 6, table S1-S3)

4. Some suggestions for modifying the figures:
Figure 2: A schematic diagram comparing the protocols of FlowMagic, Lymphoprep, and SepMate would enhance clarity and help readers understand the procedural differences.
Reply: We thank the reviewer for this valuable suggestion. We have addressed this concern by incorporating a comprehensive schematic comparison of the three protocols in Figure 2b. This diagram clearly illustrates the procedural differences between FlowMagic™, Lymphoprep, and SepMate protocols, including the key steps of density gradient centrifugation, and cell collection phases. The visual representation in Figure 2b provides readers with an immediate understanding of the methodological distinctions between these three approaches, thereby enhancing the clarity and accessibility of the comparative analysis as requested by the reviewer (Fig 2).

Figure 3: The upper, middle, and lower layers should be clearly labeled.
Reply: We thank the reviewer for this important suggestion regarding layer identification. In response to this comment, we have added clear labels for the upper, middle, and lower layers in Figure 3a, 3b, and 3c. These labels now provide precise identification of each density gradient layer, facilitating better understanding of the spatial distribution of different cell populations and debris throughout the separation process. This enhancement improves the interpretability of the results and allows readers to more effectively correlate the visual observations with the quantitative data presented in the manuscript. (Fig 3)

Figure 4 and 5: Data should include mean ± standard deviation, and statistical significance between groups (if any) should be indicated.

Reply: For the data from a total of ten volunteers presented in Figures 4, 5, and 6, the Wilcoxon signed-rank test was used with Bonferroni correction for multiple comparisons as a non-parametric alternative. All data were visualized using box plots. A significance level of 0.05 was used for all statistical tests. In response to your recommendation, we have included detailed summary tables in the Supporting Information (Tables S1-S3) that present mean values ± standard deviation and *p*-value. (Figs 4, 5, and 6, table S1-S3).

**Response to reviewer 2**

Reviewer #2: The article describes the development and evaluation of FlowMagic™, a new two-layer insert device for isolating human peripheral blood mononuclear cells (PBMCs) from whole blood, aiming to reduce red blood cell (RBC) and granulocyte (GRA) contamination commonly encountered in conventional methods. The device was compared with existing techniques (SepMate and Lymphoprep). The study addresses a significant technical challenge in the field and could be a useful tool for both research and medical laboratories, especially given the ongoing issue of RBC and GRA contamination when delayed processing is necessary due to sample transport or logistical factors.

Major Points for Improvement and Clarification:
• The experiments seem to include five volunteer donors, which is a reasonable starting point for a proof-of-concept study. However, the manuscript should clearly state whether any statistical analyses were performed, and if so, specify which tests were used, exact p-values, and confidence intervals, particularly given the sample size. This would strengthen the validity of the findings.

Specific comment:

• Was any statistical test performed for the comparisons described in the results (e.g., lines 140–141 and 148–153 regarding granulocyte contamination)? If so, please specify the tests and results in the text or figure legends.

Reply: An additional validation study was conducted using samples from five volunteers to increase the sample size to a total of 10. For the data from ten volunteers presented in Figures 4, 5, and 6, Wilcoxon signed-rank test was used with Bonferroni correction for multiple comparisons as a non-parametric alternative. All data were visualized using box plots. A significance level of 0.05 was considered statistically significant for all statistical tests. In response to this comment, we have added the above information in the figure legends. (Lines: 182-231, Figs 4 and 5.)

• While the referenced figures (e.g., Fig. 4 and 5) demonstrate improvements, including actual numerical data, summary tables in the Results section would facilitate understanding and independent assessment. Mean values ± standard deviation, exact cell counts, and percentage improvements or fold-reduction in contamination would make the benefits more explicit.

Reply: Thank you for this valuable suggestion. We agree that providing comprehensive numerical data would greatly enhance the clarity and interpretability of our findings. In response to your recommendation, we have included detailed summary tables in the Supporting Information (Tables S1-S3) that present mean values ± standard deviation, exact cell counts, and percentage improvements for all three isolation methods. These tables provide the quantitative data necessary for independent assessment of the methodological improvements and contamination reduction achieved with each approach. The inclusion of these comprehensive datasets in the Supporting Information directly addresses your concern about facilitating understanding while maintaining the flow of the main Results section. We believe these additional data will allow readers to fully evaluate the magnitude of improvements demonstrated in our comparative analysis. (Lines: 182-260, Table S1-S3)

Specific comments:
• In lines 136–138, it is stated that RBC contamination for all three methods increased after 24 and 72 hr, but Fig. 4d–f does not show an increase for FlowMagic™. How was the increase in contamination for FlowMagic™ determined? Please clarify.

Reply: We thank the reviewer for this valuable suggestion. The aforementioned description has been modified as shown below. We recognize that the previous statement may have caused confusion. At 48 and 72 hours after blood collection, the results showed an increase in the amount of RBC contamination among the two methods except FlowMagic™ (Figs 3b-c and 4b). (Lines: 182-204)

• From lines 138–140, the manuscript mentions that the FlowMagic™ method excludes RBC contamination. Please clarify how this was determined, especially in light of the previous statement about increased contamination.

Reply: We thank the reviewer for this valuable suggestion. The aforementioned description has been modified as shown below. We recognize that the previous statement may have caused confusion. Although FlowMagic™ yielded less WBC than other methods for blood samples that were incubated for 48-72 hours, it was able to exclude RBC contamination and greatly reduce granulocyte contamination (Fig. 4b). (Lines: 182-204)

• More information on the time required and ease of use of the FlowMagic™ device would be helpful. A brief comparison with the workflow of existing devices would also be valuable for readers considering adopting this technology.

Reply: We thank the reviewer for this valuable suggestion. We have addressed this concern by incorporating a comprehensive schematic comparison of the three protocols in Figure 2b. This diagram clearly illustrates the procedural differences between FlowMagic™, Lymphoprep, and SepMate protocols, including the key steps of density gradient centrifugation, and cell collection phases. The visual representation in Figure 2b provides readers with an immediate understanding of the methodological distinctions between these three approaches, thereby enhancing the clarity and accessibility of the comparative analysis as requested by the reviewer (Fig. 2).

Minor Comments:
• In line 94, the question mark should be removed, and the parenthesis with “brand” should be replaced with the actual brand name.

Reply: Thank you very much for your comments. We have already addressed this issue. (Lines: 106)

**Response to reviewer 3**

Reviewer #3: Dear Authors,
Thank you for submitting your manuscript entitled “Development of a New Cell Isolation Device FlowMagic™” for consideration. Your work presents a timely and relevant advancement in the field of peripheral blood mononuclear cell (PBMC) isolation, particularly addressing the well-known challenges of red blood cell (RBC) and granulocyte (GRA) contamination in delayed blood processing. I commend your efforts in developing the FlowMagic™ device, and your comprehensive side-by-side comparison with SepMate and standard Lymphoprep techniques is a valuable contribution to the field.
Below, I offer a detailed critique aimed at enhancing the clarity, rigour, and impact of your manuscript.

Major Comments
1. Statistical Analysis and Rigor
While your data are promising, all findings are currently presented without formal statistical analysis. This is a significant limitation. For a manuscript to meet the standards of PLOS ONE, particularly when making comparative claims, appropriate statistical testing (e.g., ANOVA with post-hoc tests, paired t-tests, or non-parametric equivalents) must be applied.

Action: Please include appropriate statistical analyses across timepoints and isolation methods for all quantitative data (e.g., cell recovery, contamination levels, flow cytometry results). Report p-values and define significance thresholds in the methods section.
Reply: An additional validation study was conducted using samples from five volunteers. For the data from a total of ten volunteers presented in Figures 4, 5, and 6, the Wilcoxon signed-rank test was used with Bonferroni correction for multiple comparisons as a non-parametric alternative. All data were visualized using box plots. A value of 0.05 was considered significant for all statistical tests. In response to your recommendation, we have included detailed summary tables in the Supporting Information (Tables S1-S3) that present mean values ± standard deviation, exact cell counts, and percentage improvements for all three isolation methods. (Figs 4, 5, and 6, table S1-S3)

2. Sample Size and Justification
The study includes five donors, but no rationale is given for the chosen sample size, and inter-donor variability is not discussed in detail.
Please provide justification for the sample size, or consider increasing the number of donors. Additionally, clarify whether the same individuals were used across the three timepoints to control for inter-subject variability.
Reply: An additional validation study was conducted using samples from five volunteers to increase the sample size to ten. For the data from a total of ten volunteers presented in Figures 4, 5, and 6, Wilcoxon signed-rank test was used with Bonferroni correction for multiple comparisons as a non-parametric alternative. All data were visualized using box plots. A significance level of 0.05 was used for all statistical tests. Parallel PBMC isolations were performed by three different techniques within 24, 48, and 72 hours after blood collection from the same volunteers. (Line: 102-103)

3. Figures 4 and 5 – Resolution and Readability
Thank you for including Figures 4 and 5, which provide critical support for your conclusions. However, both figures currently suffer from low resolution, limiting their interpretability:
Axis labels and tick marks are small and difficult to read.
No error bars or indicators of statistical variance are shown.
Legends do not consistently include units or definitions of abbreviations.
Data points are plotted but lack context (e.g., no median or confidence intervals).

Action: Please resubmit Figures 4 and 5 in high resolution (≥300 DPI). Ensure all panels include:
Clearly labeled axes (with units),
Error bars where applicable,
Statistical significance annotations (e.g., p < 0.05),
Expanded legends to enhance clarity.
Additionally, consider using consolidated bar graphs or boxplots instead of multiple dot plots, which may improve readability and facilitate comparisons.
Reply: We thank the reviewer for this valuable suggestion. We have addressed this concern through improvements in Fig 4, 5, and 6. An additional validation study was conducted using samples from five volunteers. For the data from a total of ten volunteers presented in Figures 4, 5, and 6, the Wilcoxon signed-rank test was used with Bonferroni correction for multiple comparisons as a non-parametric alternative. All data were visualized using box plots. A significance level of 0.05 was used for all statistical tests.

4. Device Limitations and Overstatements
Your discussion makes strong claims regarding the superiority and novelty of FlowMagic™. While your data are promising, such claims should be more cautiously framed unless supported by broader benchmarking or independent validation.

Please temper phrases such as “this has not been resolved until now” and consider including limitations more explicitly. For example:
Reply: We thank the reviewer for this valuable suggestion. We have already deleted the above phrase from our manuscript.

The inability of FlowMagic™ to isolate plasma simultaneously may be relevant for translational settings.
Functional downstream assays (e.g., cytokine release, ELISPOT) are not yet presented and should be acknowledged as needed future work.
Reply: We thank the reviewer for this valuable suggestion. We have already added the above phrase into the discussion section of our manuscript. (Lines: 291-300)

5. Language and Editing
The manuscript would benefit from language polishing to improve clarity, precision, and readability. There are occasional grammatical issues and ambiguous terms (e.g., “corning tubes?” on pg. 8) that should be corrected.
I recommend engaging a professional scientific editor or fluent English speaker to review the entire text.

Reply: The manuscript has been reviewed and edited by a native English speaker for language accuracy and clarity.

Minor Comments and Recommendations
Methods: Clarify if the same donor samples were used for all three timepoints across isolation methods.
Reply: We have added the following to the Materials and Methods section. Parallel PBMC isolations were performed by three different techniques within 24, 48, and 72 hours after blood collection from the same volunteers. (Line: 108-109, Fig 2)

Figures 1–2: The schematic CAD illustrations are useful; however, inclusion of dimensions would enhance technical comprehension.
Reply: We thank the reviewer for this valuable suggestion. We believe that the provided detailed images of the insert structures of the FlowMagic^TM^ isolation tube is sufficiently effective for the readers. We also believe that detailed dimensions do not affect the presentation of this technology; therefore, we have refrained from including them.

References: The citations are appropriate and up to date. However, you may wish to add references to microfluidic PBMC isolation methods, which are emerging in this space and would provide valuable context for FlowMagic™'s niche.

Reply: We thank the reviewer for this valuable suggestion. Several techniques for the separation of PBMCs utilizing microfluidics and acoustic focusing have been reported; however, these techniques are designed for microliter-level samples, which differ from the application scope of the FlowMagic™ method. Additionally, comparisons of separation performance over time post-blood collection have not been reported, making a direct comparison with the FlowMagic™ method challenging. Therefore, we would like to refrain from adding references related to microfluidic PBMC isolation at this time. We would appreciate your understanding.

References

1, Julia A, Mahdi RC, Pelle O, Anke U, Per A, Label‑free separation of peripheral blood mononuclear cells from whole blood by gradient acoustic focusing. Scientific Reports. 2024; 14: 8748.

2, Nivedita N and Ian P, Continuous separation of blood cells in spiral microfluidic devices. Biomicrofluidics. 2013 5; 7(5):054101. Doi: 10.1063/1.4819275.

3. Haotioan C, Xiaoyue K, Dan Y, Belinda DV, Johnson M, Nam-Trung N, Jun Z, High-efficient white blood cell separation from whole blood using cascaded inertial microfluidics. Talanta. 2025 284; doi.org/10.1016/j.talanta.2024.127200
